# Potential Role of Propolis in the Prevention and Treatment of Metabolic Diseases

**DOI:** 10.3390/plants10050883

**Published:** 2021-04-27

**Authors:** Georgeta Balica, Oliviu Vostinaru, Cristina Stefanescu, Cristina Mogosan, Irina Iaru, Anamaria Cristina, Carmen Elena Pop

**Affiliations:** 1 Department of Pharmaceutical Botany, Iuliu Hatieganu University of Medicine and Pharmacy, 23 Gh. Marinescu Street, 400337 Cluj-Napoca, Romania; bgeorgeta@umfcluj.ro (G.B.); cstefanescu@umfcluj.ro (C.S.); 2 Department of Pharmacology, Physiology and Physiopathology, Iuliu Hatieganu University of Medicine and Pharmacy, 6 L. Pasteur Street, 400349 Cluj-Napoca, Romania; cmogosan@umfcluj.ro (C.M.); cazacuirina16@gmail.com (I.I.); cristina.anam@yahoo.com (A.C.); 3 Department of Pharmaceutical Industry, Iuliu Hatieganu University of Medicine and Pharmacy, 12 I. Creanga Street, 400010 Cluj-Napoca, Romania; carmen.pop@umfcluj.ro

**Keywords:** propolis, antidiabetic, antihyperlipidemic, anti-obesity

## Abstract

Propolis is a resinous mixture with a complex chemical composition, produced by honeybees and stingless bees from a variety of vegetal sources. In the last decades, propolis was extensively researched, multiple studies confirming its anti-inflammatory, antioxidant, antimicrobial, and wound-healing properties. More recently, due to an exponential increase in the number of patients with metabolic diseases, there is also a growing interest in the study of antidiabetic, antihyperlipidemic, and anti-obesity effects of propolis. The aim of this review was to evaluate the potential role of propolis in the prevention and treatment of metabolic diseases like diabetes mellitus, dyslipidemia, and obesity. The preclinical in vivo and in vitro pharmacological models investigating antidiabetic, antihyperlipidemic, and anti-obesity effects of propolis were reviewed with a focus on the putative mechanisms of actions of several chemical constituents. Additionally, the available clinical studies and an evaluation of the safety profile of propolis were also presented.

## 1. Introduction

Propolis, commonly known as the “bee glue”, is a natural resinous mixture produced by honeybees (mostly *Apis mellifera*) and some other bees, such as stingless bees from resinous and gummy substances gathered from leaves, buds, sap flows, trichomes, and other actively exuding plant structures. Honeybees take the vegetable materials with their mandibles and mix them with some salivary enzymes like alpha-amylase, beta-amylase, maltase, or some esterases. Other bees, such as stingless bees species produce propolis by collecting resinous material from plants and mixing it with beeswax and soil to form the so called geopropolis. Bees are using propolis to protect hives by blocking the cracks, sealing the spaces, and smoothing out the internal walls to maintain a constant inner temperature and to attain an internal aseptic environment [1,2,3,4,5].

Bee products have been used since ancient times as important bioresources because of their widely beneficial properties. Egyptians, Greeks, and Romans reported the biological properties of propolis for lesion healing. Aristotle, Dioscorides, Pliny, and Galen described some of the medicinal properties of propolis and they used propolis as antiseptic and mouth disinfectant as well as for healing wounds. In the medieval period, these applications of propolis were spread by Arabian physicians. The Incas used propolis as an antipyretic. Since the 18th century, propolis was first included in the London Pharmacopeia as an official drug. Between, the 17th and 20th centuries, propolis became popular in Europe, due to its antibacterial activity. During the Second World War, propolis was used as an antimicrobial and anti-inflammatory agent [6,7].

The World Health Organization estimates that between 70% and 95% of the population from developing countries use natural products as a therapeutic alternative [6]. Nowadays, propolis is available in the form of capsules, as an extract, as a mouthwash, in throat lozenges, creams, and in powder form as well as part of cosmetic formulations and health food items [7]. The wide application of propolis in modern medicine is due to its diverse pharmacological and biological properties, such as antibacterial, antifungal, antiviral, antiprotozoal, antioxidative, spasmolytic, astringent, anti-inflammatory, anesthetic, antitumor, immunostimulant, and hepatoprotective properties [1,2,3,8].

Recently, due to an exponential increase in the number of patients with metabolic diseases which could lead to significant or even fatal cardiovascular complications, propolis was also tested in vivo and in vitro for antidiabetic, antihyperlipidemic, or anti-obesity effects, with promising results. The aim of this review was to present the preclinical, clinical, and safety data linked to the potential role of propolis in the prevention and treatment of metabolic diseases, highlighting also the available mechanistic studies on individual chemical constituents.

## 2. Materials and Methods

This study is a review of available scientific data concerning the effects of propolis in the prevention and treatment of metabolic diseases. A search was performed in Web of Science, PubMed, and Scopus scientific databases, including the last twenty years. The search terms “propolis”, “antidiabetic” (“hypoglycemic”), “dyslipidemia” (“antihyperlipidemic”), and “obesity” were used for data selection. Only full-text articles in English were included in this work. Our study identified 54 preclinical and clinical studies, a total of 30 studies being selected after the removal of duplicates and materials written in other languages.

## 3. Types of Propolis and Chemical Composition

The botanical source of propolis is represented by several different plants distributed all over the world. Exudates from poplar buds (*Populus* spp.) are described as the main botanical source of propolis from temperate regions, as well as birch (*Betula alba* L.), horse chestnut (*Aesculus hippocastanum* L.), alder (*Alnus glutinosa* Medik), beech (*Fagus sylvatica* L.), and some conifers [9,10]. In propolis samples from different tropical regions, the main plant sources are *Baccharis dracunculifolia* D.C., *Araucaria angustifolia* (Bertol.) Kuntze, *Clusia minor* L., *Clusia rosea* Jacq., *Dalbergia ecastophyllum* (L.) Taub., *Macaranga tanarius* (L.) Müll. Arg., *Hyptis divaricata* Pohl. ex. Benth, and *Eucalyptus citriodora* (Hook) [10,11]. The most important bee products, such as honey and pollen are referred to the botanical name of the plant from which they originate [2]. Based on the plant source and the area of collection, propolis has been categorized into seven types: poplar, birch, Brazilian green, Brazilian red, Clusia, Pacific, and Mediterranean [12,13]. Propolis composition is extremely complex and variable, showing the presence of 50–55% resin, 30% beeswax, 10–15% essential oils, and 5% pollen [3,14,15]. Physical properties, such as color, aroma, and consistency of propolis depend on factors like geographic origin, types of vegetal sources, time of collection, and season of the year [1,2]. The color varies from yellow, green to red, and dark brown [4,15]. Propolis has a typical odor and a bitter taste [16]. The odor can vary from sample to sample, having a distinct flavor and an aromatic pleasant smell, some samples being odorless [15,16,17]. This complex mixture has variable consistency, it is hard and brittle when cold but becomes soft and very sticky when warm [16].

The chemical composition of propolis strongly depends on geographical location. Botanical origins and chemical composition of propolis have a close relationship leading to great variation in constituents of the propolis [18]. Extensive research was conducted over the years on different molecules identified in the composition of propolis and until 2018 more than 850 compounds were reported, 305 of them isolated for the first time between 2013 and 2018. As propolis samples from unexplored areas (mainly in Africa or Asia) are being analyzed, the number of constituents is constantly increasing, new compounds being reported in studies published between 2018 and 2021, such as new flavanones and phenantrendiol derivatives in African samples, and new prenylflavonoids in Asian samples [19,20,21].

Generally, a propolis sample contains in average 80–100 different constituents [4]. The specific compounds of the propolis are phenolic compounds (flavonoids as main constituents, phenolic acids, and their esters, phenylpropanoids), terpenes and terpenoids, ketones, aromatic aldehydes and alcohols, proteins, fatty acids, waxy acids, amino acids, hydrocarbons, steroids, stilbenes, sugars, vitamins, minerals, and enzymes. The phenolic compounds are believed to be the most representative biologically active constituents of propolis, especially in poplar type. They were found to represent on average around 28% (±9%) of the whole mass of poplar propolis, among which 8% (±4%) are flavones/flavonols and 6% (±2%) are flavanones/dihydroflavonols [4]. Other compounds, such as the flavonoid glycosides, alkaloids, and tannins were discovered recently [1,4,22]. The main chemical compounds from propolis are presented in Table 1.

For each of the seven types of propolis, several compounds are specific, although the phenolic compounds (flavonoids, aromatic acids, and their esters) are characteristic for the propolis obtained from temperate regions (Europe, Asia, North America) and terpenoids for that obtained from tropical (Brazil, Africa) and Mediterranean regions [35,37,41,42,43].

Poplar propolis is produced in temperate zones and the main botanical sources are the bud exudates of *Populus* species, mostly *Populus nigra* L. [4,9,42,47]. It is composed mainly of flavonoids (chrysin, galangin, pinocembrin, pinobanksin, pinobanksin-3-*O*-acetate, pinocembrin chalcone, quercetin, kaempferol, apigenin, naringenin), phenolic acids, and their esters, sesquiterpenes [36,37,48,49,50,51,52].

Birch propolis is produced in Russia from birch buds and it consists mainly of flavones, flavonols, flavonones, and sesquiterpenes [1,4,47,48].

Brazil green propolis, the most popular tropical propolis type, is originate from the leaves of *Baccharis dracunculifolia* D.C. Recently, Brazil red propolis that originates from red resinous exudates at the surface and the branch orifice of *Dalbergia ecastophyllum* (L.) Taub [4,43] was discovered. Green and red propolis are the most common of the 13 types of Brazilian propolis and are composed of prenylated phenylpropanoids (specifically artepillin C, baccharin, drupanin in green propolis), phenolic acids, p-coumaric acids, diterpenic acids, kaempferide, apigenin, isosakuranetin, and typically for red propolis, formononetin, isoliquiritigenin, biochanin A, daidzein, vestitol [21,26,33,34,44,48].

Another tropical propolis type, Clusia propolis or Cuban red propolis is the one originating from resin exuded by the flowers of different *Clusia* species found in Cuba and Venezuela [4,52]. It is rich in isoflavones, isoflavanes, flavonoids, and isoprenylated benzophenones [31,47].

Pacific propolis or Taiwanese green propolis found in Taiwan, Okinawa, and Indonesia originates from the fruits of *Macaranga tanarius* (L.) Müll. Arg. [4,53]. This type of propolis is characterized by the presence of prenylated flavonoids (propolins, prokinawan, nymphaeol, isonymphaeol) [33,47,53].

The specificity of Mediterranean propolis, that seems to originate from cypress, is the high concentration of terpenoids (mainly totarol and diterpenic acids: isocupressic, communic, pimaric, imbricatoloic, abietic acids). This type is found in Greece, Malta, Sicily, Turkey, and Algeria [4,28,37,42,47,52]. If this propolis is obtained solely of cypress trees, the extract does not contain flavonoids, nor phenolic acids, but only diterpenes totarol and totarolon at high concentrations [50].

In the last decades, propolis has gained extensive popularity as a functional food and dietary supplement. In order to extract propolis for commercial purposes, ethanol, glycerol, and water are the main solvents employed, other solvents being also available. Ethanol is currently the most used solvent to obtain low wax propolis extracts rich in biologically active compounds. Recently, new methods of extracting propolis have been studied in order to replace the conventional ethanolic extraction method. One of the most promising methods is supercritical fluid extraction, which has the capacity to retain the antioxidant properties of the obtained propolis extracts through its use of low temperatures, which is an important characteristic for the pharmaceutical and food industries [54].

## 4. Preclinical Studies Investigating the Effects of Propolis in Metabolic Diseases

Our review identified 22 preclinical in vivo and in vitro studies, which were focused on the investigation of the effects of propolis in metabolic diseases like diabetes mellitus, dyslipidemia, or obesity (Table 2).

The in vivo animal models used to evaluate antidiabetic effect of propolis used streptozotocin (STZ), alloxan, D-glucose, or fructose to induce specific pathological modifications in glucose metabolism leading to chronic hyperglycemia, a key factor associated with cardiovascular complications. In these studies, the administration of propolis reduced the rise of blood glucose and ameliorated insulinemia with protective effects on pancreatic beta cells in chemically induced diabetes mellitus [55,56,57,58]. Additionally, several in vitro studies demonstrated inhibitory effects of propolis on several enzymes involved in glucose metabolism (alpha-glucosidase, maltase, or alpha-amylase), with the reduction of digestive absorption of glucose, which may also contribute to the overall antidiabetic effect [59,60,66].

For the evaluation of the effects of propolis in dyslipidemia, the majority of the in vivo experimental models used a high-fat diet in order to induce an increase in the serum concentration of cholesterol and triglycerides and only one model used sodium nitrate to induce hypercholesterolemia [68]. Another experimental approach was to use genetically engineered animals like APOE2 or LDL r-/- transgenic mice, which develop severe dyslipidemia due to alterations of enzymes and receptors involved in cholesterol metabolism [70,71,72]. In all experiments, the administration of propolis decreased the concentration of total cholesterol, LDL, and triglycerides. Additionally, propolis proved to be protective against the development of aortic lesions and arterial atherogenesis in transgenic animals [71].

The experimental models used to study the anti-obesity effect of propolis used C57BL/6J mice, in which the weight gain was diet induced. The treatment with propolis caused a reduction of body weight gain and an increased thermogenesis in adipose tissue, also reducing the accumulation of visceral adipose tissue [75,76].

## 5. Clinical Studies Investigating the Effects of Propolis in Metabolic Diseases

The effects of propolis in metabolic diseases were investigated in eight clinical studies focused on diabetes, obesity, or diabetic complications like diabetic foot ulcer (Table 3).

In diabetic patients there is a significant risk of macrovascular or microvascular complications with a high mortality rate and impaired quality of life. Several randomized controlled studies (Afsharpour et al., 2019; Zakerkish et al., 2019; Samadi et al., 2017) proved that oral administration of propolis for at least 2 months in diabetic patients caused a reduction of fasting blood glucose (FBG) and glycosilated hemoglobin (HbA1C), which are considered important predictors of vascular complications, therefore showing a significant protective effect in diabetes mellitus [77,78,79]. However, other clinical studies (Zhao et al., 2016; Fukuda et al., 2015) did not show a significant influence on glucose level itself in diabetic patients treated with propolis but demonstrated a reduction of oxidative stress and inflammation, with favorable consequences in long-term management of diabetes mellitus [80,81]. The differences between clinical studies could be caused by multiple factors like variations of the doses used in patient treatment, a different geographical origin of propolis with a subsequently modified chemical composition or differences in study design and surveyed outcomes.

Only one clinical study (Natsir et al., 2020) evaluated the effect of propolis in a small cohort of patients with central obesity, proving a reduction of leptin level, without assessing other endpoints [82].

Additionally, the randomized controlled studies of Mujica et al., 2019 and Henshaw et al., 2014 demonstrated a significant effect of cutaneously applied propolis in the treatment of an important diabetic complication, the diabetic foot ulcer, the obtained results showing an accelerated wound healing in treated patients [83,84].

The presented clinical studies have some limitations, being represented by small scale randomized placebo controlled trials (RCTs) using a reduced number of enrolled patients. Therefore, larger clinical trials with a superior statistical significance are needed in order to warrant a possible clinical use of propolis in diabetes mellitus, its complications, or other metabolic diseases.

## 6. Mechanistic Studies with Active Constituents from Propolis in Metabolic Diseases

### 6.1. Inhibition of Alpha-Amylase and Alpha-Glucosidase in Diabetes Mellitus

Alpha-amylase and alpha-glucosidase are digestive enzymes necessary for the breakdown of complex molecules like starch or maltose to glucose, which can be absorbed into the bloodstream and subsequently used as energy source. The most important of the two enzymes is alpha-glucosidase, situated on the brush border of the small intestine which is capable of hydrolyzing disaccharides to alpha-glucose. The inhibition of alpha-glucosidase can decrease the glucose absorption and finally the amount of glucose in the bloodstream [85].

**Figure 1:**
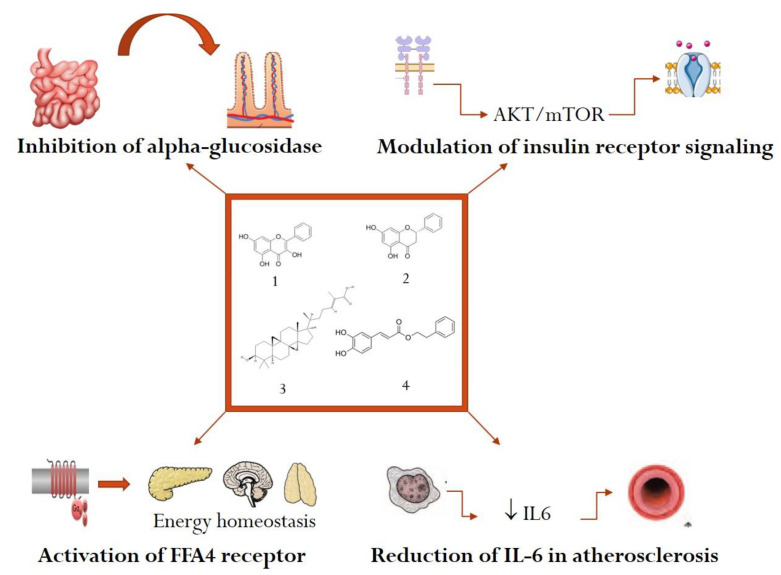
Examples of mechanisms of action of selected active constituents from propolis in metabolic diseases (1—galangin, 2—pinocembrin, 3—mangiferonic acid, 4—CAPE).

Several drugs with inhibitory effects on alpha glucosidase can mitigate postprandial hyperglycemic peaks, being useful in the treatment of type 2 diabetes. However, multiple adverse effects like abdominal cramps or diarrhea could reduce patient adherence to treatment [85], therefore natural products with alpha glucosidase inhibitory effect may become successful drug candidates for the management of diabetes mellitus. The study of Pujirahayu et al., 2019 tested the inhibitory effect of several triterpenes from propolis (cycloartenol, ambonic acid, mangiferonic acid, and ambolic acid) on alpha-glucosidase. The results showed that **mangiferonic acid** from propolis had the strongest inhibitory effect on alpha-glucosidase with an IC50 of 3.46 µM/mL (Figure 1) [86].

### 6.2. Modulation of Insulin Receptor Signaling in Diabetes Mellitus

Insulin receptor signaling leading to the translocation of glucose transporters on the membrane of hepatocytes, adipocytes, and skeletal muscle cells is a key process involved in the regulation of glucose, lipid, and energy metabolism [87]. The modulation of insulin receptor signaling in different steps of the intracellular pathway can augment the response to insulin in several types of tissues and subsequently reduce insulin resistance [87].

Several insulin receptor signaling modulators of natural origin have been tested with promising results. The research of Liu et al., 2018 proved that two important chemical constituents from propolis, galangin, and pinocembrin modulated insulin receptor signaling acting via Akt/mTOR pathway. The two compounds reduced insulin resistance by upregulating IR, Akt, and GSK3β and downregulating the phosphorylation of IRS. It is known that in diabetes the serine/threonine phosphorylation of IRS may cause a reduction of insulin signal transduction, therefore the intracellular effect of **galangin** and **pinocembrin** from propolis can restore insulin receptor sensitivity and alleviate insulin resistance, as shown in Figure 1 [88].

Additionally, the study of Nie et al., 2017 showed that **caffeic acid phenethyl ester (CAPE)** present in the chemical composition of propolis was able to enhance p-Akt, inhibiting simultaneously p-JNK, amplifying insulin effects at receptor level with a reduction of insulin resistance in diabetic mice [89].

### 6.3. Anti-Inflammatory Mechanisms in Dyslipidemia and Atherosclerosis

Recently, atherosclerosis is increasingly regarded as an inflammatory condition at vascular level, an inhibition of inflammatory processes being considered a valuable strategy to reduce the progression of endothelial lesions. Hence, IL-6, a pro-inflammatory cytokine produced mainly by macrophages can favor the development and progression of atherosclerosis. In humans, a clinical trial (Bacchiega et al., 2017) proved that IL-6 is a major player in the inflammatory events leading to atherosclerosis and the blockade of this cytokine with specific inhibitors like tocilizumab may reduce cardiovascular risk, unfortunately with significant adverse reactions [90]. Other cytokines like IL-17 have inhibitory roles, the study of Simon et al. proving that elevated levels of IL-17 are associated with better outcomes in patients with myocardial infarction, due to atherosclerosis [91].

Propolis proved to be a significant inhibitor of IL-6 in experimental models of inflammation and, in addition, the study of Fang et al., 2013 proved that propolis decreased the level of IL-6 while increasing IL-17 in a rodent model of dyslipidemia and atherosclerosis [71]. A previously published study of Bachiega et al., 2012 showed that **cinnamic and coumaric acids** from propolis significantly inhibited IL-6 production in macrophages from BALB/c mice [92].

### 6.4. Antioxidant Mechanisms in Dyslipidemia

A series of in vitro and in vivo experimental models have shown that oxidative stress is directly involved in the pathogenesis of atherosclerosis, the life-threatening consequence of dyslipidemia. In the vascular wall, oxidized low density lipoproteins are internalized in macrophages with the formation of foam cells which promote cell proliferation and endothelial dysfunction. In hypercholesterolemic animals, atherosclerotic processes were favored by the generation of reactive oxygen species which induced an increased oxidation of LDL [72].

Propolis has a high content of antioxidant molecules, being able to decrease lipid peroxidation and the generation of reactive oxygen species with positive effects on the cardiovascular system. Silva et al., 2015 demonstrated that propolis was able to prevent left ventricular hypertrophy (LVH) and atherogenesis in hypercholesterolemic mice, due to its ability to eliminate superoxide and hydroxyl radicals and the reduction of CD40L expression [70].

The chemical constituents from propolis responsible for the antioxidant effect are considered to be polyphenols and flavonoids, present in all types of propolis in different concentrations, influenced by plant origin, bee species, temperature, or geographic factors. The study of Kocot et al., 2018 identified specific propolis compounds like **3,5-dicaffeoylquinic acid**, **artepillin C** or **3,4,5-tricaffeoylquinic** acid as being responsible for the antioxidant effect [93].

### 6.5. Activation of FFA4 Receptor with Positive Effects in Obesity

Obesity is a complex and multifactorial disease, which can lead to an inflammatory condition triggered by the toll-like receptor 4 (TLR4), with a role in the etiology of cardiovascular diseases [94]. The inflammatory response from obesity can be mitigated by some unsaturated fatty acids like eicosapentaenoic acid (EPA) or docosahexaenoic acid (DHA), which are agonists on the free fatty acid receptor 4 (FFA4). This G-protein coupled receptor is present in the enteric nervous system but also in adipocytes, pancreas, or brain, being involved also in the regulation of energy homeostasis, appetite control, or adipocyte differentiation. The activation of FFA4 receptor leads to a reduction of the kinase activated by the growth factor beta (TAK1) activity and consequently an inhibition of IKK-β/NF-kβ and JNK/AP-1 pathway with anti-inflammatory consequences [94].

The study of Cho et al., 2020 found that several phenolic constituents from propolis like **pinocembrin**, **chrysin**, and **galangin** were able to activate the FFA4 receptor in vitro. **Pinocembrin**, a flavanonic compound from propolis, was the most potent activator of FFA4 receptor with potential applications in the pharmacological management of obesity and its complications [95].

## 7. Safety Profile of Propolis

Propolis is a natural product that is widely accepted by patients at a time when natural products are increasingly popular, to the detriment of chemically produced drugs. Although several in vivo studies in animals and humans aimed at demonstrating certain therapeutic effects of propolis were performed, they were not focused on the determination of adverse effects and toxicity, as propolis is generally recognized as safe (GRAS) [96]. Most of the chemical constituents in propolis are harmless and well tolerated if the doses are not too high. It is estimated that ingestion of 70 mg propolis/day is potentially non-toxic for the organism, however, exceeding the dose of 15 g/day may cause adverse effects [15,97]. The major compounds in propolis belong to the class of polyphenols (flavonoids, phenolic acids, and their esters). It is considered that except for caffeic acid phenethyl ester (CAPE), all other polyphenols have a low order of acute oral toxicity, but the toxicity of individual compounds was rarely tested. A study showed that galangin, an important chemical constituent from propolis, had no toxicity in doses up to 320 mg/kg in Wistar rats [98]. Pinocembrin, another active constituent from propolis was found to be non-toxic and non-mutagenic in doses up to 100 mg/kg in rats [99].

In humans, the occurrence of adverse effects following the administration of propolis has been observed both in oral and in local administration to the skin or throat. As a direct result of topical application of cosmetic and pharmaceutical formulations, adverse reactions included dermatitis, urticaria, swelling, and ulcerative gingivitis, especially in atopic patients. The study of Walgrave et al., 2005, reported that 1.2–6.6% of dermatitis patients were sensitive to propolis [100]. In general, adverse reactions were moderate, but literature also mentioned cases of patients with anaphylactic shock with laryngeal edema induced by the local administration of propolis [101]. The studies aimed to assess the allergenic potential of propolis have revealed that the major allergen in propolis is LB-1, which is a mixture of three isomeric pentenyl caffeates [15,101].

Despite the numerous studies focused on its chemical composition and beneficial effects, a chemical standardization of propolis is necessary, in order to be officially accepted into the mainstream of health care systems. However, due to its complex and variable chemical composition, it is difficult to find universally valid criteria. Literature mentions Bankova’s approach, which considers that a quantification by group of structurally related compounds is more appropriate [102]. Future studies will continue the efforts to standardize propolis, for a safer and more effective administration.

## 8. Conclusions

Our review identified 22 preclinical and 8 clinical studies, which proved a series of favorable effects of propolis in diabetes mellitus, dyslipidemia, and obesity. Inhibition of alpha-glucosidase, modulation of insulin receptor signaling, reduction of IL-6, and activation of FFA4 receptors were the most important mechanisms of action identified for several chemical constituents from propolis: galangin, pinocembrin, mangiferonic acid, and CAPE. Additional studies are needed to ascertain the importance of propolis as a useful functional food for the prevention and treatment of metabolic diseases.

## Figures and Tables

**Table 1:** Main chemical compounds present in the composition of honeybees and stingless bees propolis.

Flavonoids	apigenin, kaempferol, pinobanksin, chrysin, tektochrisin, pinocembrin, galangin, quercetin, myricetin, rutin, rhamnetin, isorhamnetin, luteolin, naringenin, acacetin, baicalein, hesperitin, sakuranetin, formononetin, liquiritigenin, isalpinin, daidzein, genistein, eupatorin, hispidulin, propolins, prokinawan, isosativan, medicarpin, vestitol, nymphaeol, isonymphaeol [1,18,23,24,25,26,27,28,29,30,31,32,33]
Phenyl carboxylic acids and derivatives	caffeic acid, caffeic acid phenethyl ester, cichoric acid, cinnamic acid, ferulic acid, *p*-coumaric acid, benzoic acid, salicylic acid, rosmarinic acid, chlorogenic acid, caffeoylquinic acid, vanillic acid, artepillin C, baccharin, drupanin [18,22,27,29,33,34,35,36,37,38]
Terpenoids	geraniol, nerol, bisabolol, guaiol, farnesol, linalool, limonene, eudesmol, terpineol, camphor, squalene, copaene, calarene, calamenene, caryophyllene, patchoulene, elemene, ferruginol, junicedric acid, pimaric acid, abietic acid, isocupressic acid, acetylisocupressic acid, communic acid, imbricatoloic acid, totarol, amyrin, amyrone, lupeol, lupenone, moretenol, ferutinin, teferin, germanicol, agarospirol, lanosterol, erythrodiol, cycloartenol, ambonic acid, mangiferonic acid, ambolic acid [1,6,22,28,31,34,35,37,39,40,41,42,43,44]
Amino acids	aspartic acid, glutamic acid, serine, glycine, histidine, arginine, threonine, alanine, proline, tyrosine, valine, methionine, isoleucine, leucine, phenylalanine, lysine, tryptophane, asparagine, cystine [1,45]
Aliphatic hydrocarbons and aliphatic acids	eicosine, 1-octadecene, eicosane, heneicosane, docosane, tricosane, tetracosane, pentacosane, hexacosane, heptacosane, octacosane, nonacosane, triacontane, behenic acid, cerotic acid, lauric acid, linoleic acid, lignoceric acid, montanic acid, nonanoic acid, palmitic acid, oleic acid, stearic acid, behenic acid, decanoic acid, dodecanoic acid, tetradecanoic acid, heptadecanoic acid, tetracosanoic acid, eicosanoic acid, hexacosanoic acid [1,34,43,46]
Sugars and sugar alcohols	xylose, galactose, mannose, glucuronic acid, lactose, maltose, melibiose, d-ribofuranose, d-fructose, d-gulose, talose, sucrose, d-glucose, erytritol, xylitol, inositol, d-glucitol [1,33]
Vitamins	B1, B2, B3, B5, B6, C, E [1,4,45]
Minerals	Sr, Ba, Cd, Sn, Pb, Ti, Ag, Co, Mo, Al, Si, V, Ni, Mn, Cr, Na, Mg, Cu, Ca, Zn, Fe, K [1,33]
Alkaloids	demecolcine, papaverine, thebaine, morpholine, norlobeline, pagicerine, oreophilin [4,34,44]

**Table 2:** Preclinical studies (in vivo and in vitro) investigating the effect of propolis in metabolic diseases.

No.	Experimental Model/Dose of Propolis (In Vivo)	Findings	Reference
	Antidiabetic effect		
1.	d-glucose induced diabetes in rats/100–200 mg/kg	Reduction of fasting blood glucose; reduction of insulin resistance; reduction of body weight	Laaroussi et al., 2020 [55]
2.	Streptozotocin induced diabetes in rats/300 mg/kg	Reduction of fasting blood glucose	Nna et al., 2019 [56]
3.	Streptozotocin induced diabetes in rats/50–100 mg/kg	Reduction of blood glucose; reduction of serum creatinine and urea	El Menyiy et al., 2019 [57]
4.	Streptozotocin induced diabetes in mice/300 mg/kg	Reduction of blood glucose	Rivera-Yanez et al., 2018 [58]
5.	In vitro assessment of alpha-glucosidase	Inhibition of alpha-glucosidase with IC50 of 70.79 ± 6.44 µg/mL	Vongsak et al., 2015 [59]
6.	In vitro assessment of alpha-glucosidase and α-amylase	Inhibition of alpha-glucosidase with IC50 of 0.01 ± 0.01 mg/mL; inhibition of alpha-amylase with IC50 of 0.09 ± 0.01 mg/mL	Popova et al., 2015 [60]
7.	Alloxan induced diabetes in rats/200–300 mg/kg EO	Reduction of blood glucose; conservation of normal pancreatic cell architecture	Babatunde et al., 2015 [61]
8.	Streptozotocin induced diabetes in rats/100 mg/kg	Reduction of fasting blood glucose; reduction of glycated hemoglobin; restoration of STZ-altered hepatorenal functions	Zhu et al., 2013 [62]
9.	Streptozotocin induced diabetes in rats/200 mg/kg	Reduction of serum glucose; reduction of oxidative stress parameters	El Sayed et al., 2009 [63]
10.	Fructose induced diabetes in rats/100–300 mg/kg	Reduction of plasma level of insulin and HOMA-R index of insulin resistance	Zamami et al., 2007 [64]
11.	Alloxan induced diabetes in rats/1 mL/100 g	Reduction of blood glucose; reduction of fructosamine, malonaldehyde and nitric oxide	Fuliang et al., 2005 [65]
12.	In vitro assessment of maltase and α-amylase	Inhibition of maltase with IC50 of 1.0 mg/mL; inhibition of alpha-amylase with IC50 of 4.7 mg/mL	Matsui et al., 2004 [66]
	**Antihyperlipidemic effect**		
13.	High-fat diet mice/50 mg/kg	Reduction of total cholesterol and triglycerides; reduction of atherogenic index of plasma	Orsolic et al., 2019 [67]
14.	Sodium nitrite induced hyperlipidemia in guinea pigs/200 mg/kg	Reduction of cholesterol, triglycerides; reduction of atherogenic index of plasma	Azab et al., 2015 [68]
15.	High-fat diet rats/1–2% *w*/*w*	Reduction of cholesterol, triacylgycerol and ALT	Albokhadaim, 2015 [69]
16.	LDL r-/- mice/70 µL/animal	Increase of plasmatic HDL; prevention of LVH and arterial atherogenesis	Silva et al., 2015 [70]
17.	ApoE-knockout mice/160 mg/kg	Reduction of total cholesterol, triglycerides, and non-HDL; decrease atherosclerotic lesion development in aortic root	Fang et al., 2013 [71]
18.	LDL r-/- mice/250 mg/kg	Normalisation of lipid profile/downregulation of VCAM, FGF, VEGF and MMP-9 gene expression	Daleprane et al., 2012 [72]
19.	High-fat diet rabbits/75 mg/kg	Reduction of total cholesterol, LDL and triglycerides	Nader et al., 2010 [73]
20.	High-fat diet rats/0.05–0.5% *w*/*w*	Reduction of cholesterol and triglycerides/increase of PPARα protein level in the liver	Ichi et al., 2009 [74]
	**Anti-obesity effect**		
21.	Obese C57BL/6J mice	Increased thermogenesis in white adipose tissue (WAT); activation of creatine metabolism pathways	Nishikawa et al., 2020 [75]
22.	Obese C57BL/6J mice/5–50 mg/kg	Reduction of body weight gain; down-regulation of fatty acid synthase and SREBP mRNA expression	Koya-Miyata et al., 2009 [76]

**Table 3:** Clinical studies with propolis administered in metabolic diseases.

Disease	Type of Clinical Study	Number of Patients	Treatment/Dose	Results	Reference
Diabetes mellitus	Randomized, placebo-controlled study	62 patients with type 2 diabetes	1500 mg/day propolis, orally for 8 weeks	Reduction of HbA1C; increase of TAC blood levels and activity of GPx and SOD	Afsharpour et al., 2019 [77]
Diabetes mellitus	Randomized, placebo-controlled study	50 patients with type 2 diabetes	1000 mg/day propolis, orally for 90 days	Reduction of HbA1C, HOMA-IR, hs-CRP	Zakerkish et al., 2019 [78]
Diabetes mellitus	Randomized, placebo-controlled study	66 patients with type 2 diabetes	900 mg/day propolis, orally for 12 weeks	Significant reduction of FBG and HbA1C; decrease of total cholesterol	Samadi et al., 2017 [79]
Diabetes mellitus	Randomized controlled study	32 patients with type 2 diabetes	900 mg/day propolis, orally for 18 weeks	Reduction of carbonyls, LDH activity and TNFα	Zhao et al., 2016 [80]
Diabetes mellitus	Randomized, placebo-controlled study	80 patients with type 2 diabetes	226.8 mg/day propolis, orally for 8 weeks	Prevention of eGFR worsening; limited impact on HOMA-IR	Fukuda et al., 2015 [81]
Obesity	Randomized, placebo-controlled study	30 patients with central obesity	60 mg/day propolis, orally for 2 weeks	Reduction of leptin level	Natsir et al., 2020 [82]
Diabetic foot ulcer	Randomized controlled study	31 patients with diabetic foot wounds	Cutaneously applied propolis	Reduction of wound area, accelerated healing; reduced TNFα	Mujica et al., 2019 [83]
Diabetic foot ulcer	Prospective, controlled study	24 patients with diabetic food ulcer	Cutaneously applied propolis	41% reduction of ulcer area; accelerated wound healing	Henshaw et al., 2014 [84]

## Data Availability

Not applicable.

## Abbreviations

ALT	aspartate aminotransferase
APOE2	apolipoprotein E2
FFA4	free fatty acid receptor 4
FGF	fibroblast growth factor
HDL	high-density lipoprotein
HOMA-R	homeostasis model assessment for insulin resistance
IC50	half-maximal inhibitory concentration
IRS	insulin receptor substrate
LDL	low-density lipoprotein
LVH	left ventricular hypertrophy
MMP-9	matrix metalloproteinase 9
mTOR	mammalian target of rapamycin
PPAR	peroxysome proliferator activated receptor
SREBP	sterol regulatory element binding protein
STZ	streptozotocin
VCAM	vascular cell adhesion molecule-1
VEGF	vascular endothelial growth factor
